# Case report: Personalized triple phage-antibiotic combination therapy to rescue necrotizing fasciitis caused by Panton-Valentine leukocidin-producing MRSA in a 12-year-old boy

**DOI:** 10.3389/fcimb.2024.1354681

**Published:** 2024-09-17

**Authors:** Brieuc Van Nieuwenhuyse, Mathilde Balcaen, Olga Chatzis, Astrid Haenecour, Emilien Derycke, Thierry Detaille, Stéphan Clément de Cléty, Cécile Boulanger, Leïla Belkhir, Jean-Cyr Yombi, Julien De Greef, Olivier Cornu, Pierre-Louis Docquier, Audrey Lentini, Renaud Menten, Hector Rodriguez-Villalobos, Alexia Verroken, Sarah Djebara, Maya Merabishvili, Johann Griselain, Jean-Paul Pirnay, Laurent Houtekie, Dimitri Van der Linden

**Affiliations:** ^1^ Institute of Experimental and Clinical Research, Pediatric Department (IREC/PEDI), Université catholique de Louvain - UCLouvain, Brussels, Belgium; ^2^ Pediatric Intensive Care Unit, Cliniques universitaires Saint-Luc, Université catholique de Louvain – UCLouvain, Brussels, Belgium; ^3^ Pediatric Infectious Diseases, General Pediatrics Department, Cliniques universitaires Saint-Luc, Université catholique de Louvain - UCLouvain, Brussels, Belgium; ^4^ Genetics of Autoimmune Diseases and Cancer laboratoire (GEDI), de Duve Institute, Université catholique de Louvain – UCLouvain, Brussels, Belgium; ^5^ Department of Pediatric Hematology and Oncology, Cliniques Universitaires Saint-Luc, Université catholique de Louvain - UCLouvain, Brussels, Belgium; ^6^ Division of Internal Medicine and Infectious Disease, Cliniques Universitaires Saint-Luc, Université catholique de Louvain - UCLouvain, Brussels, Belgium; ^7^ Louvain centre for Toxicology and Applied Pharmacology, Institute of Experimental and Clinical Research (IREC/LTAP), Université catholique de Louvain - UCLouvain, Brussels, Belgium; ^8^ Department of Orthopedic and Trauma Surgery, Cliniques Universitaires Saint-Luc, Université catholique de Louvain - UCLouvain, Brussels, Belgium; ^9^ Institute of Experimental and Clinical Research, Neuromusculoskeletal Lab (IREC/NMSK), Université catholique de Louvain - UCLouvain, Brussels, Belgium; ^10^ Departement of Plastic Surgery, Cliniques universitaires Saint-Luc, Université catholique de Louvain – UCLouvain, Brussels, Belgium; ^11^ Department of Radiology, Pediatric Radiology Unit, Cliniques universitaires Saint-Luc, Université Catholique de Louvain – UCLouvain, Brussels, Belgium; ^12^ Department of Microbiology, Cliniques universitaires Saint-Luc, Université catholique de Louvain - UCLouvain, Brussels, Belgium; ^13^ Institute of Experimental and Clinical Research, Medical Microbiology Department (IREC/MBLG), Université catholique de Louvain - UCLouvain, Brussels, Belgium; ^14^ Center for Infectious Diseases, Queen Astrid Military Hospital, Brussels, Belgium; ^15^ Laboratory for Molecular and Cellular Technology, Queen Astrid Military Hospital, Brussels, Belgium

**Keywords:** MRSA, Panton and Valentine leukocidin (PVL), bacteriophage, phage therapy, necrotizing fasciitis, pediatric intensive care unit

## Abstract

Maximal standard-of-care (SOC) management could not stop the life-threatening progression of a necrotizing fasciitis induced by Panton-Valentine Leukocidin-producing Methicillin-Resistant *Staphylococcus aureus* (MRSA) in a 12-year-old boy. Multi-route phage therapy was initiated along with antibiotics against *Staphylococcus aureus, Pseudomonas aeruginosa* and *Stenotrophomonas maltophilia*, eventually leading to full recovery with no reported adverse events.

## Introduction

*S. aureus* is an important pathogen of necrotizing fasciitis, and MRSA isolates in particular have been reported to feature increasing prevalence and high correlated mortality in these patients (Cheng et al., 2011; Tsai et al., 2021). Panton-Valentine Leukocidin (PVL) is a cytotoxin produced by certain *S. aureus* strains, and is known to enhance *S. aureus*’ virulence and necrosis-inducing potential; accordingly, necrotizing fasciitis resulting from PVL-*S. aureus* infection have been reported (Gillet, 2013; Hussain et al., 2022). In this case, PVL-MRSA-induced necrotizing fasciitis failed to respond to maximal SOC management: as a last resort therapy, it was treated by an exceptional multi-route phage therapy (PT) specifically targeting three involved bacterial species, in combination with antibiotics. This did not trigger any detected adverse event, and was followed by complete resolution of the infection.

## Case description

**Figure 1:**
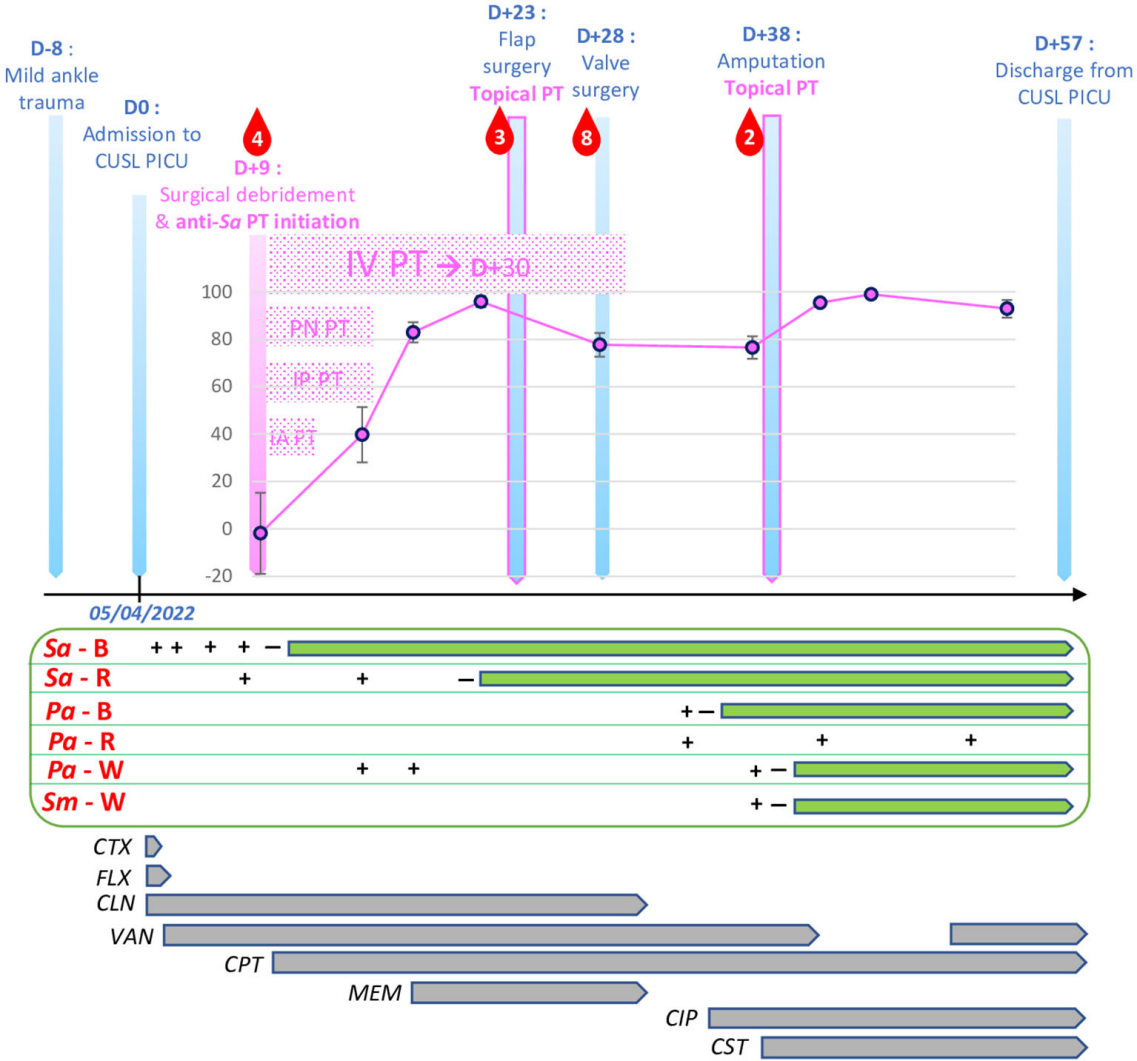
Timeline of the clinical case's main events. Main medico-surgical events are represented as blue vertical arrows. Phage therapy-related events are represented in pink. Pink curve represents degree of Phage Immune Neutralization (PIN) against phage ISP in % of neutralized titer at 30 minutes. Error bars represent standard deviation of the means. Red droplets represent units (205 mL) of blood transfused. D , day; Sa, Staphylococcus aureus; Pa, Pseudomonas aeruginosa ; Sm, Stenotrophomonas maltophilia ; B, Blood culture ; R, Respiratory tract-originated sample culture (broncho-alveolar lavage or endotracheal aspirate) ; W, wound sample culture (swab or intraoperative biopsy); PT, phage therapy; IV, intravenous; PN, pulmonary nebulization; IP, intra-pleural; IA, intra-abdominal; CTX, Cefotaxime; FLX, Flucloxacillin; CLN, Clindamycin; VAN, Vancomycin; CPT, Ceftaroline; MEM, Meropenem; CIP, Ciprofloxacin; CST, Colistin.

A 12-year-old boy with no significant medical or surgical history was transferred (D0) to the Pediatric Intensive Care Unit (PICU) (**Figure 1**). On D-8, he suffered a minor trauma causing a sprained left ankle with superficial scratches. A plaster boot cast was set on D-7, but removed on D-6 because the patient reported increasing pain. On D-3, the child became confused and on D-1, febrile at 39°C. Clinical findings included tachycardia, polypnea, and swollen, painful, warm and red left leg and foot. Biological findings included signs of inflammatory syndrome (C-reactive protein 304 mg/L - normal value <5 mg/L) and multiple organ failure including respiratory and hemodynamic failure as well as liver, kidney and muscle damage, leading to PICU admission. Blood cultures were performed and empirical intravenous (IV) flucloxacillin, clindamycin and cefotaxime were initiated. Exceeding intramuscular pressure was measured in all eight tested compartments in the left foot and leg. Fasciotomies were performed, revealing pus in the soft tissues of the left lower limb.

Vasopressor therapy, sedation and endotracheal intubation were maintained upon return to PICU. Microbiological cultures of pus and blood grew for methicillin-resistant *Staphylococcus aureus* (MRSA) producing Panton-Valentine Leukocidin (PVL). IV vancomycin was initiated on D+1, replacing cefotaxime and flucloxacillin, while clindamycin was pursued. Since we suspected significant contribution from the PVL toxin to the patient’s condition, IV polyclonal immunoglobulins (1 g/kg) were administered on D+1.

Despite these adapted standard-of-care (SOC) measures and in-range therapeutic drug monitoring of vancomycin within 48h of initiation, the infection rapidly progressed to generalized necrotizing fasciitis and pyomyositis on D+2, as confirmed by full-body MRI (**Figure 1**). This justified extended fasciectomies and debridement on multiple sites on D+8. Ascites appeared and was drained on D+2. Thoracic CT-scan revealed disseminated septic emboli in both lungs on D+1. Chest X-ray showed a right pleural effusion on D+8, which was drained with purulent discharge confirming empyema. Microbiological cultures of both the peritoneal and pleural fluids grew for the same MRSA-PVL strain.

## Diagnosis, therapeutic measures, outcomes

**Figure 2:**
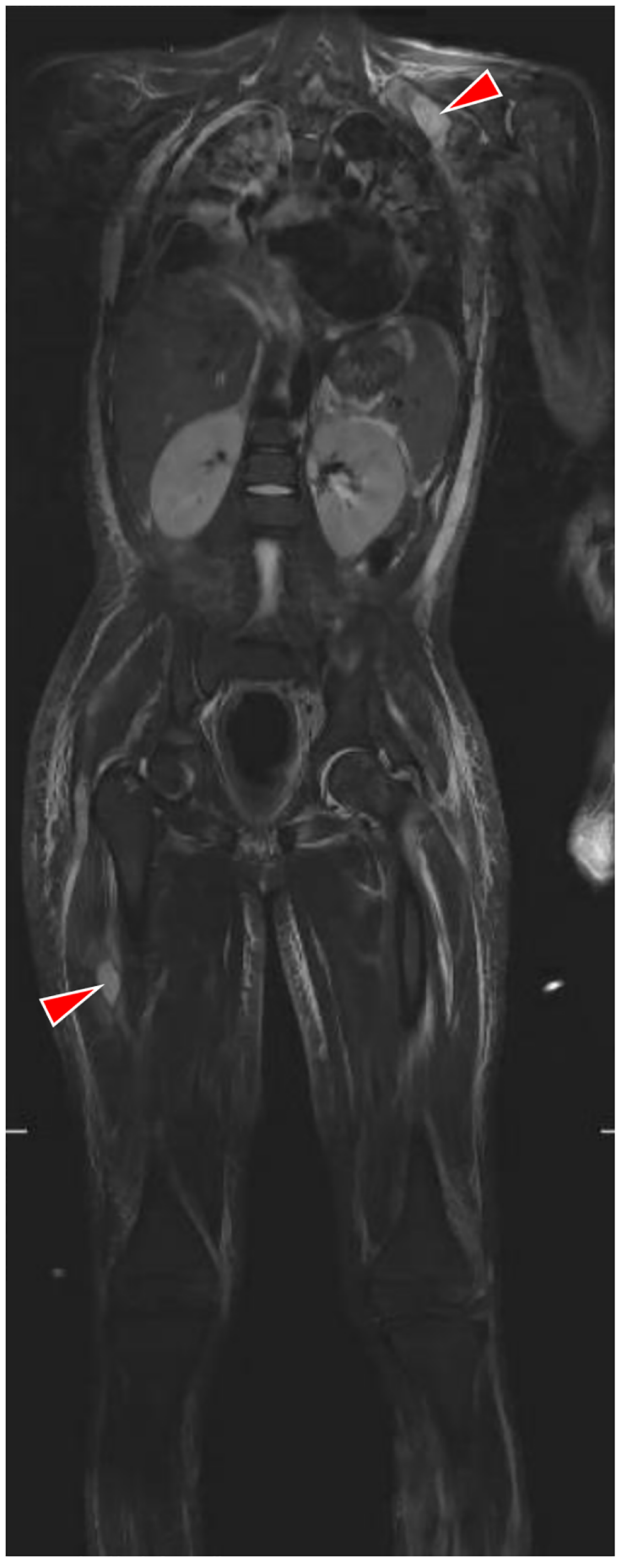
Full body MRI, coronal view. Short-TI inversion recovery (STIR) mode. Image shows diffuse hyperintense inflammatory infiltration in subcutaneous soft tissues of both lower limbs, the upper left limb, the thorax and the abdomen, as well as disseminated inflammatory pulmonary lesions and two inflammatory fluid collections in the right thigh and left shoulder (red arrows).

Between D0 and D+8, sustained MRSA-PVL bacteremia was observed and the patient’s state was significantly deteriorating to a life-threatening situation, despite maximal SOC management. Necrotizing fasciitis diagnosis was assessed based on magnetic resonance imaging (MRI) findings (**Figure 2**), microbiological culture findings (*S. aureus-*positive pus and blood cultures), and aforementioned evidence of compartment syndrome by multiple compartment pressure measurements. *S. aureus-*positive blood cultures combined with persistent need for vasopressor therapy defined the complementary diagnosis of septic shock syndrome.

As rescue therapy, phage therapy (PT) was initiated on D+9. PT is the use of specific bacteriophage viruses (“phages”), natural viruses that exclusively infect and lyse a specific bacterial genre or species, as bactericidal therapeutic agents. A solution containing the anti-*S. aureus* phage named ISP was obtained from Queen Astrid Military Hospital ([QAMH], Brussels, Belgium) after on-plate phage susceptibility testing (“phagogram”) revealed that phage ISP had potent lytic power against the patient’s MRSA-PVL strain (EOP = 1, i.e. “efficiency of plating”, a phage’s relative lytic efficiency on a clinical strain compared to the phage’s reference host strain, expressed as a ratio of the number of plaques theoretically ranging from 0 to 1). The initial ISP phage solution, of titer 10^9^ plaque forming units (PFU)/mL, was diluted 100 times in NaCl 0,9% to reach a therapeutic titer of 10^7^ PFU/mL. Multi-route PT was then initiated using this 10^7^ PFU/mL solution (**Figure 1**). IV PT was administered at 50mL over 6h every 24h during 22 days (D+9 to D+30). Intra-pleural and intra-peritoneal PT were administered through the respective drainage catheters: once daily, cavities were rinsed with 5% sodium bicarbonate solution for 15 minutes, before 40mL of phage solution were instilled. Catheters were then clamped for 20 minutes before being reopened, without aspiration. Topical PT was applied daily on open wounds with dressings soaked in phage solution for 20 minutes. Lastly, bronchopulmonary PT was administered by mesh-nebulization of 4 mL of phage solution at the higher titer of 10^8^ PFU/mL. IV ceftaroline was also added to the treatment eight hours after the first IV PT dose. Additionally, following isolation of *Pseudomonas aeruginosa* in several wounds on D+14, IV meropenem was administered from D+17 until D+30 (**Figure 1**).

Objective improvements were rapidly noticed. The first blood culture performed after PT initiation and ceftaroline initiation (sampled on D+10, i.e. 30h after PT initiation and 22h after ceftaroline initiation) did not grow for MRSA-PVL, nor did any ulterior blood culture. Peritoneal and pleural catheters were withdrawn after three and seven days of PT respectively. Despite these improvements, the evolution remained complicated, with extubation failure related to persistent lung damage and bacterial tracheitis, and mitral valve abscess related to sustained MRSA-PVL bacteremia, cured by open-heart valve repair surgery on D+28. Septic necrosis led to extensive tissue loss in the left foot and leg, with no hope for spontaneous wound closure. Reconstruction by flap surgery using right latissimus dorsi was attempted on D+23. Intra-operative topical PT was applied using ISP solution (10^7^ PFU/mL) for syringe rinsing of the surgical site and soaking of dressings applied for 45 minutes. However, flap necrosis started on D+32, eventually leading to transtibial amputation of the left leg on D+38. Infection of the left leg wounds by *Stenotrophomonas maltophilia* and *P. aeruginosa* had been diagnosed on D+37. Accordingly, we administered intra-operative topical PT on the leg stump before surgical closure, applying soaked dressings and rinsing with 200 mL of a 10^7^ PFU/mL mix of anti-*S. maltophilia* phage BUCT700 and anti-*P. aeruginosa* phages PNM, 14-1 and PT07. Like anti-*S. aureus* phage ISP, these anti-*S. maltophilia* and anti-*P. aeruginosa* phages were also selected based on favorable phage susceptibility testing (“phagogram”) results on the respective patient’s bacterial isolates.

After amputation, sustained favorable clinical progression was observed along durable eradication of all *S. aureus*, *S. maltophilia* and *P. aeruginosa* from the bloodstream and all wounds. Besides amputation, the child progressively reached full recovery of all wounds and of respiratory function. The child was transferred from PICU to pediatric hospitalization unit on D+57, then to rehabilitation unit on D+104. Final discharge from hospital occurred on D+145. One year after hospital discharge, the patient remains cured of all infections. Stump osteitis was suspected on clinical basis 5 months after hospital discharge, and was successfully treated by surgical revision along with vancomycin and clindamycin administration. Functional rehabilitation and adaptation to the prosthetic leg appear successful. Cardiological follow-up upon initial hospital discharge diagnosed ectopic atrial tachycardia, likely linked to previous bacterial valve abscess and related surgery. This was initially treated by flecainide, which was successfully withdrawn after 4 months without needing further re-introduction.

## Discussion

This severe infection required an exceptional multiple bacteriophage-antibiotic combination therapy directed against three pathogens: *S. aureus, P. aeruginosa* and *S. maltophilia*. This is likely the first report ever of a patient being treated by three distinct documented phage therapies, targeting three distinct pathogens, all with their dedicated route(s) of administration and combined antibiotic therapy. Sustained MRSA bacteremia and its associated metastatic sites of infection are a known therapeutic challenge: seemingly appropriate SOC management often fails to eradicate bacteremia, and PT is increasingly reported as a potentially promising complementary approach (Holland et al., 2022). In this case, PT initiation was followed by the eradication of these three pathogens in their respectively treated compartments. Yet, several aspects of the clinical and microbiological progression deserved further attention.

The occurrence of Phage Immune Neutralization (PIN), the development of an antibody response against administered phages, was investigated using modified Adams protocol by double agar overlay assay on numerous serum samples collected before, during and after PT (Adams, 1959). PIN occurred against phage ISP, starting six days after PT initiation (D+14) (**Figure 1**). Transient decrease in PIN magnitude between D+21 and D+41 is likely due to hemodilution by numerous blood transfusions (**Figure 1**) and extracorporeal circulation during open-heart surgery. Consistent with previous studies, this potent and early-onset PIN was not followed by therapeutic failure, though it might mitigate the efficacy of ulterior reintroduction of phage ISP (Łusiak-Szelachowska et al., 2017; Dedrick et al., 2021).

Evaluation of PT’s net contribution to *S. aureus’* eradication from the bloodstream is a limitation to this work. It is indeed difficult because of the initiation of ceftaroline eight hours after PT initiation and the absence of blood cultures that could have been collected in this short interval between PT initiation and ceftaroline initiation.

**Figure 3:**
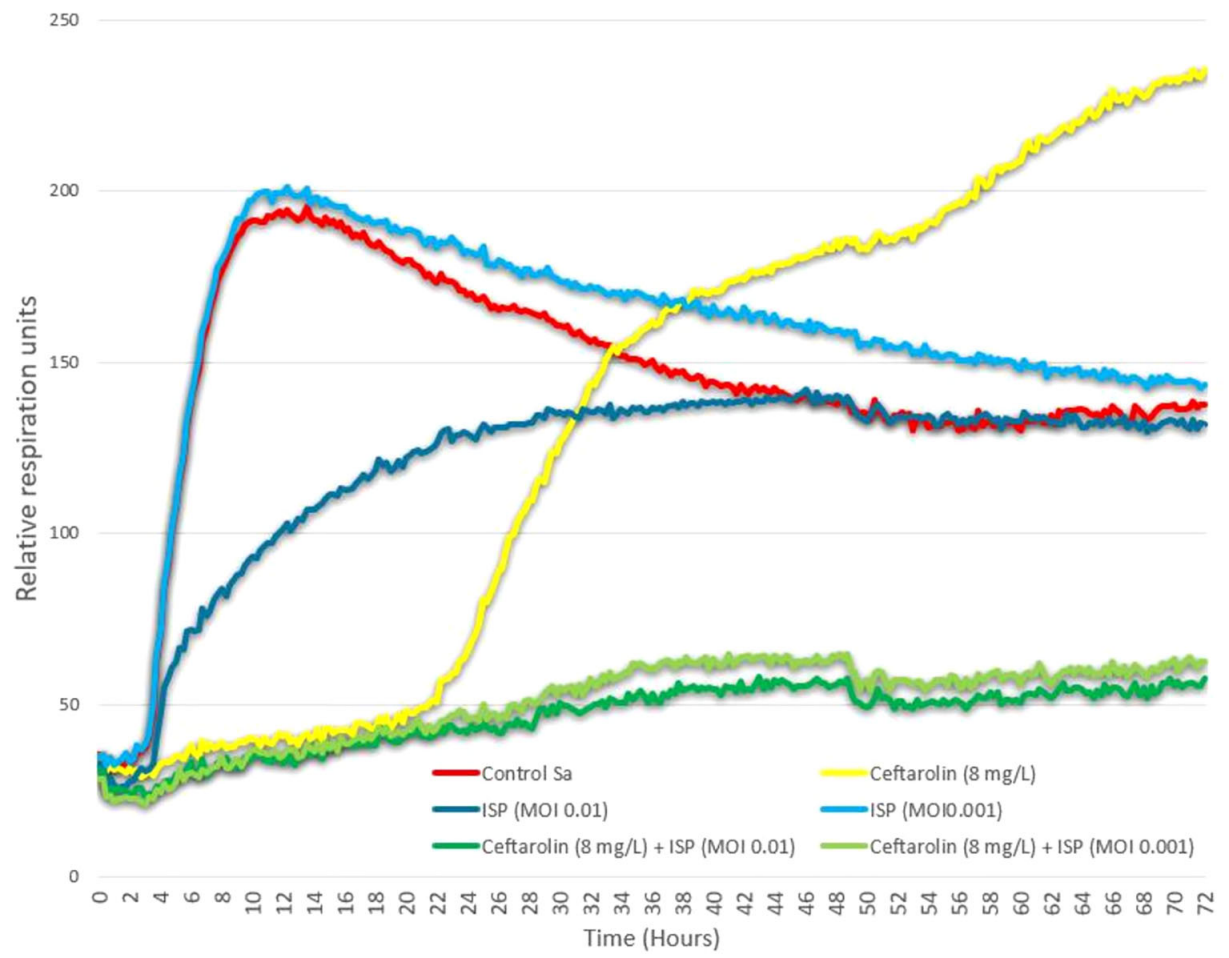
Phage-antibiotic interaction testing. Activities of phage ISP (at multiplicity of infection [MOI] = 0.01 and 0.001) and ceftaroline (at 8mg/L) as well as the combinations of these phage titers with this ceftaroline concentration, were determined using an OmniLog® automated incubator (Biolog, Hayward, CA, USA). Data was analyzed with OmniLog data Analysis Software (v1.7). Experiments were done in 96-well plates in a final volume of 200 µl of LB supplemented with 100 times diluted tetrazolium dye mix, according to the manufacturer’s instructions. Bacterial cells were added at a concentration of 105 colony forming units(cfu)/well, calculated based on optical density (OD, at 600 nm) measurements (with an OD of 0.5 corresponding to 4 x 108 cfu/ml, on average), which were validated using a classical plate culture method. Plates were incubated at 37 ºC for 72 hours and a possible reduction (causing a color change) of the tetrazolium dye due to bacterial respiration (during growth) was monitored and recorded every 15 min by the Omnilog system. Bacterial proliferation is presented through relative units of cellular respiration : efficacious phages, antibiotics, and combinations thereof, suppress bacterial proliferation. "Control Sa" curve represent control growth of a triplicate containing only the aforementioned bacterial load (S. aureus), with no antibiotic or phage. Results are presented as mean values of biological triplicates. While ceftaroline alone at 8mg/L and phage ISP alone at two different MOIs appear unable to durably suppress bacterial growth over the 72 hours of growth analysis, combination of this ceftaroline concentration with either of these two sub-inhibitory phage titers leads to durable suppression of bacterial growth, suggesting in vitro synergistic properties.

Nevertheless, using an OmniLog^®^ automated incubator, we investigated and compared in 96-wells microplates triplicates the *in vitro* bacterial growth kinetics of the patient’s *S. aureus* strain subjected to various combinations of different titers of phage ISP, ceftaroline, and the combination of both, as already reported in similar works (Van Nieuwenhuyse et al., 2021, 2022). The results from this assay suggest, at least at certain phage and antibiotic titers, that their combined used is able to durably suppress bacterial growth in a synergistic fashion (**Figure 3**). The translatability of these *in vitro* properties to *in vivo* conditions, however, warrants further research.

PVL is a cytotoxin produced by various *S. aureus* strains, both methicillin-sensitive *S. aureus* (MSSA) and community-acquired methicillin-resistant *S. aureus* (CA-MRSA) (Shallcross et al., 2013). It is known to enhance *S. aureus*’ virulence and necrosis-inducing potential (Gillet, 2013).

Some observations in our work correlated with features typically described in PVL-*S. aureus* infections: first, our patient had a severe infection leading to PICU as a result of hemodynamic and respiratory failure from a septic shock. In a prospective European multicentric study, 17% of children with community-acquired S. aureus (CA-SA) invasive infections had severe infection leading to death or admission to PICU, caused by both CA-MSSA and CA-MRSA strains (Gijón et al., 2016). The prevalence of PVL-positive CA-SA invasive infections amounted to 18.6%, and only 7.8% of the isolates were MRSA. In this study, PVL expression is a factor independently associated with outcome severity, regardless of methicillin resistance (Gijón et al., 2016).

Second, PVL-associated bone and joint infections (BJI) in children are correlated with higher biological inflammatory markers at presentation, a longer course of illness, more febrile days, more complicated/severe infection (muscle abscess, pyomyositis, subperiosteal abscess, visceral abscess, and deep venous thrombosis) and more intensive care unit admissions, according to a systematic review including 15 studies on children suffering from such BJI (Bouiller and David, 2023). These typical features are again in line with our report.

Third, PVL-associated community-acquired pneumonia (CAP) and its severity are not evenly distributed according to age. Gillet and colleagues described two distinct entities of PVL-associated staphylococcal CAP with differences regarding clinical presentation and outcome: staphylococcal CAP in toddlers (i.e. pleuropneumonia) and staphylococcal necrotizing pneumonia in young adults. In toddlers, PVL-negative CAP is virtually absent, presentation matches with pleuropneumonia, SOC appears to be sufficient and overall mortality is low (Gillet, 2013; Gillet et al., 2021). Contrastingly, necrotizing PVL-associated CAP in adolescents and adults is associated with specific symptoms at admission as cutaneous rash, airway hemorrhage and leukopenia. It occurs mostly in younger, previously healthy adults, and is often preceded by influenza-like symptoms. Its outcomes are extremely severe with rapid onset of acute respiratory distress syndrome (ARDS) despite aggressive management, accounting for high mortality (39%) (Sicot et al., 2013; Gillet et al., 2021). In our case, despite PVL status and absence of underlying conditions, the entity of staphylococcal necrotizing pneumonia was not entirely congruent because the patient showed no airway bleeding, no leukopenia, and had a delayed onset of pneumonia. A secondary pulmonary involvement due to secondary hematogenous infection and septic pulmonary emboli seems more likely, as opposed to an inhalation-mediated infection leading to the aforementioned typical necrotizing phenotype.

Lastly, sub-MIC titers of β-lactams and, to a lesser extent, vancomycin may enhance bacterial production of PVL, but this over-expression can be blocked by the combined use of an antitoxin agent (Dumitrescu et al., 2008). As such, the concomitant use of clindamycin, rifampin or linezolid is thus recommended in clinical practice (Dumitrescu et al., 2008). Nevertheless, antibiotics alone often fail to fully contain such infections, as antibiotic distribution is reduced in necrotic tissue. Therefore, early and complete drainage of all PVL suppuration and debridement of necrotic tissues by extensive and, if needed, repeated surgeries is mandatory whenever feasible and as soon as possible (Gillet et al., 2011). This additional need for surgical management in bone and joint infections and complicated skin and soft-tissue infections is again consistent with our case.

It should also be noted that further PCR-based typing of the *S. aureus* strain revealed the absence of expression of exfoliative toxins ETA and ETB and of toxic shock syndrome toxin 1 (TSST-1) (**Table 1**). Protein A (spa)-typing linked the strain to type t5691, related to multilocus sequence type ST152 (Lebughe et al., 2017). ST152 is a known community-acquired MRSA-PVL. Its spread in both Europe and sub-Saharan Africa is consistent with the patient’s history (Baig et al., 2020). However, ST152 is not known as an intrinsically virulent clone (Baig et al., 2020). The triggering of such a hyper-virulent disseminated infection by a non-hyper-virulent MRSA-PVL strain led to the research of specific risk factors, including inborn errors of immunity (IEI) (Gillet, 2013; Bousfiha et al., 2022). Routine immunological work-up showed no abnormal values in blood count except for low B-cells (143/µL [N: 200-600/µL]), more marked in the B-memory cells (Total B-memory CD27+ cells: 14/µL [N: 50-200/µl] – relative abundance 9.8% [N: 13.3-47.9%]). Whole-exome sequencing provided no molecular basis allowing for the diagnosis of IEI, and specifically no heterozygous defects in OTULIN, which are known to expose carriers to severe necrotic staphylococcal infections by dysregulating nonhematopoietic cells’ response to alpha-toxin, a major staphylococcal virulence factor (Spaan et al., 2022) (**Supplementary Tables 1** and **2**).

**Table 1:** Strain typing.

S. aureus	typing	*S. aureus*	AST		*P. aeruginosa*	AST	*S. maltophilia*	AST
		antibiotic	MIC	interp.	antibiotic	interp.	antibiotic	interp.
PVL	positive	Amikacin	8	R	Amikacin	S	Amikacin	R
TSST-1	negative	Ceftaroline	1	S	Aztreonam	S	Aztreonam	R
ETA	negative	Clindamycin	<0.25	S	Ceftazidim	S	Ceftazidim	R
ETB	negative	Ciprofloxacin	<0.25	S	Ciprofloxacin	S	Ciprofloxacin	S
spa-type	t5691	Cefoxitin	>8	R	Colistin	S	Cefepim	R
MST type	ST152	Gentamycin	>4	R	Cefepim	S	Gentamycin	R
		Moxifloxacin	<0.25	S	Imipenem	S	Imipenem	R
		Oxacillin	>2	R	Minocyclin	R	Minocyclin	S
		Rifampin	<0.25	S	Meropenem	S	Meropenem	R
		TMP-SMX	>4/76	R	Piperacillin-Taz.	S	Piperacillin-Taz.	R
		Teicoplanin	<1	S			TMP-SMX	S
		Tetracyclin	<0.5	S				
		Vancomycin	1	S				

Antibiotic susceptibility testing (AST) data are presented for an array of tested antibiotics for aforementioned isolates of S. aureus, P. aeruginosa and S. maltophilia. For S. aureus, minimum inhibitory concentrations (MIC) were assessed by standard microdilution method, expressed in mg/L. AST is assessed by standard disk diffusion method for P. aeruginosa and S. maltophilia. Interpretation (interp.) finds the respective isolates susceptible (S) or resistant (R) to the tested antibiotic. Further characterization of typical virulence factors of S. aureus is performed by polymerase chain reaction (PCR) and illustrates expression of PVL (Panton-Valentine Leukocidin) by the isolate, but no expression of TSST-1 (toxic shock syndrome toxin 1) or exfoliative toxins A or B (ETA, ETB). Further protein- and genome-based characterization of the S. aureus isolate links it to spa-type t5691, related to multilocus sequence type ST152. TMP-SMX, trimethoprim-sulfamethoxazole; piperacillin-taz., piperacillin-tazobactam.

Whole-genome sequencing of the initially retrieved S. aureus isolate was performed to refine resistance and virulence typing. Genomic DNA was extracted (DNeasy UltraClean Microbioal kit, Qiagen) and prepared for Nextera Flex (Illumina) and sequenced on an Illumina Miniseq machine using a paired-end approach (2*150 bp). The quality of the sequencing data was assessed (Fast QC, Galaxy Version 0.12.1) and trimmed appropriately (Trimmomatic (Bolger et al., 2014), Galaxy Version 0.39). Genomes were constructed using Unicyler (Galaxy Version 0.5.0) (Wick et al., 2017). Genome annotation was performed using Prokka (Galaxy Version 1.14.6) (Seemann, 2014). Further function annotation was performed using Abricate (Galaxy Version 1.0.1) (Seemann, 2017) through ARG-ANNOT (ARG-ANNOT NT v.6, July 2019), CARD (v.3.1.4 to 3.2.5), ResFinder, https://bitbucket.org/genomicepidemiology/resfinder_db) for antibiotic resistance genes and VirulenceFinder 2.05,6 database for virulence genes (accessed on July 8, 2024) (Joensen et al., 2014; Tetzschner et al., 2020). The genomes were scanned against PubMLST schemes using MLST7 (Galaxy Version 2.22.0).

Sequencing product is available on NCBI BioProject with accession number JBFQYI000000000. Results confirmed the findings from both the former first-line molecular typing and the antibiotic susceptibility testing. Indeed, regarding antimicrobial resistance, presence of genes *dfrG, blaZ*, and *aac(6’)-aph(2”)* were phenotypically correlated to aforementioned resistance to trimethoprim-sulfamethoxazole, (amino) penicillins and aminosides respectively, while *mecA* was responsible for the identified MRSA phenotype and its associated resistance to all tested Beta-lactam antibiotics besides ceftaroline. These sequencing products were used in SCCmecFinder (version 1.2) to predict the strain’s SCCmec type. No whole SCCmec cassette was predicted with a template coverage threshold of at least 40%. Nevertheless, SCCmec type XIII (9A) was considered the most likely prediction based on mec class A and ccr class 9 (93.1% identity query with ccrC2 allele 1:1:KR187111), SCCmec type XIII having been first characterized in 2018 based on another ST152 strain (Baig et al., 2018). Results regarding virulence confirmed the presence of genes *lukF-PV* and *lukS-PV* coding for Panton Valentine leukocidin F & S components respectively, and initial annotation attempt did not detect any known enterotoxin or exfoliatin present in the used database. These synthetic results are made available as **Supplementary Material**. However, a specific BLAST confirmed that this strain expressed a variant of exfoliative toxin E (coded by *ete* gene) with 100% homology to an *ete* variant, called *ete2*, recently described in a case report which was interestingly also focusing on a PVL-positive clonal complex 152 *S. aureus-*related necrotizing fasciitis (Sabat et al., 2022). In this work, the authors also point out that *ete2* variant has been described exclusively in clonal complex 152 *S. aureus* so far. The net contribution of *ete2* to the virulent phenotype encountered in these necrotizing fasciitis cases is still unclear, and warrants further research. These synthetic results are made available as **Supplementary Table 3**.

## Patient perspective

Adolescents may be able to make medical decisions on their own, even when the choice and its implications are particularly challenging. However, in this case, direct discussion with the patient at the most critical time during the course of events was not possible due to sedation and intubation during the septic shock management. It was thus not possible to define if he was mature enough to understand the situation in full. Subsequently, exposing the full complexity of the situation to his parents, and more specifically convincing them eventually that transtibial amputation was essential to save their son’s life was no easy task. It required sustained day-to-day communication about this complex situation, making sure every aspect of it was understandable while avoiding the pitfall of over-simplification; most of all, it mostly required deep empathy at all time. Despite the heartbreaking nature of this decision, the parents eventually consented to the transtibial amputation, convinced it was indeed the only way to stop further progression of this life-threatening infection.

## Data Availability

The datasets presented in this study can be found in online repositories. The names of the repository/repositories and accession number(s) can be found in the article/**Supplementary Material**.

## Supplementary material

Supplementary Table 1 Patient genome - sequencing findings in the field of OTULIN & OTULINL genes modifications.

Supplementary Table 2 Patient genome - whole-exome sequencing and annotation data.

Supplementary Table 3 Strain genome - sequencing and annotation data in the field of antimicrobial resistance and virulence determinants.
